# An Evaluation of BfmR-Regulated Antimicrobial Resistance in the Extensively Drug Resistant (XDR) *Acinetobacter baumannii* Strain HUMC1

**DOI:** 10.3389/fmicb.2020.595798

**Published:** 2020-10-29

**Authors:** Candace M. Marr, Ulrike MacDonald, Grishma Trivedi, Somnath Chakravorty, Thomas A. Russo

**Affiliations:** ^1^Department of Medicine, University at Buffalo, Buffalo, NY, United States; ^2^Erie County Medical Center, Buffalo, NY, United States; ^3^Veterans Affairs Western New York Healthcare System, Buffalo, NY, United States; ^4^Department of Microbiology and Immunology, University at Buffalo, Buffalo, NY, United States; ^5^Witebsky Center for Microbial Pathogenesis, University at Buffalo, Buffalo, NY, United States

**Keywords:** *Acinetobacter baumannii*, meropenem, polymyxin E, extensively drug resistant, biofilm, BfmR, MapA, OprB

## Abstract

*Acinetobacter baumannii* is a problematic pathogen due to its common expression of extensive drug resistance (XDR) and ability to survive in the healthcare environment. These characteristics are mediated, in part, by the signal transduction system BfmR/BfmS. We previously demonstrated, in antimicrobial sensitive clinical isolates, that BfmR conferred increased resistance to meropenem and polymyxin E. In this study, potential mechanisms were informed, in part, by a prior transcriptome analysis of the antimicrobial sensitive isolate AB307-0294, which identified the porins OprB and aquaporin (Omp33-36, MapA) as plausible mediators for resistance to hydrophilic antimicrobials such as meropenem. Studies were then performed in the XDR isolate HUMC1, since delineating resistance mechanisms in this genomic background would be more translationally relevant. In HUMC1 BfmR likewise increased meropenem and polymyxin E resistance and upregulated gene expression of OprB and aquaporin. However, the comparison of HUMC1 with isogenic mutant constructs demonstrated that neither OprB nor aquaporin affected meropenem resistance; polymyxin E susceptibility was also unaffected. Next, we determined whether BfmR-mediated biofilm production affected either meropenem or polymyxin E susceptibilities. Interestingly, biofilm formation increased resistance to polymyxin E, but had little, if any effect on meropenem activity. Additionally, BfmR mediated meropenem resistance, and perhaps polymyxin E resistance, was due to BfmR regulated factors that do not affect biofilm formation. These findings increase our understanding of the mechanisms by which BfmR mediates intrinsic antimicrobial resistance in a clinically relevant XDR isolate and suggest that the efficacy of different classes of antimicrobials may vary under biofilm inducing conditions.

## Introduction

*Acinetobacter baumannii* is a nosocomial pathogen that has plagued healthcare facilities by its capacity to survive for prolonged periods on abiotic surfaces and express extensive drug resistance (XDR). This ability is, in part, mediated by the two-component sensor/response signal transduction system BfmS/BfmR. BfmR is the transcriptional response regulator that propagates the signal from its corresponding sensor protein BfmS. This response regulator oversees a number of crucial phenotypes including enhanced biofilm production (Tomaras et al., 2008), virulence, and antibiotic resistance (Tomaras et al., 2008; Russo et al., 2016). Our group previously demonstrated that in the antimicrobial sensitive clinical isolates *A. baumannii* AB307-0294 and AB908, BfmR mediated increased resistance to meropenem and polymyxin E (colistin), but not to tigecycline, thereby excluding a generalized increase in permeability (Russo et al., 2016). Other investigators, using the ATCC strain 17978, demonstrated that BfmS/R modulates resistance to imipenem (Liou et al., 2014; Geisinger et al., 2018). Therefore, the central role of BfmS/R in antimicrobial resistance has raised interest in assessing BfmR or BfmR-regulated gene products as possible drug targets (Thompson et al., 2012; Russo et al., 2016; Draughn et al., 2018). However, it would be important to demonstrate these findings in an XDR clinical isolate.

An important step for translating these data into clinical practice is the delineation of the relative roles of BfmR-regulated gene products in conferring resistance. This information is needed for the prioritization of targets for downstream development of new therapeutic modalities. We hypothesized that the intrinsic antimicrobial resistance mediated by BfmR is multifactorial and may direct unique mechanisms of resistance for different antimicrobial classes.

Therefore, to define the genes in the BfmR regulon that contributed to intrinsic antimicrobial resistance, we utilized generated transcriptomic data (RNA-Seq) that compared AB307-0294 (wt) and its BfmR-deficient derivative AB307.70 (Δ*bfmR*). A bioinformatics analysis and expression levels were used to filter putative gene products that could contribute to BfmR-regulated antimicrobial resistance to meropenem and polymyxin E. These data served as a starting point to identify potential contributory genes in an XDR isolate since for the optimal translation of preclinical data, it is important to study clinical strains with the greatest translational impact (Russo et al., 2016; Wong et al., 2017). Therefore, the XDR *A. baumannii* strain HUMC1 was chosen for further study (Luo et al., 2012).

A significant contributor to *A. baumannii’s* intrinsic antibiotic resistance is its general impermeability to all substances, including antimicrobials. This decreased permeability is in part mediated by porins. In *A. baumannii*, porins comprise only about 5% of its outer membrane proteins by mass, compared to 60% in *E. coli* (Vila et al., 2007). Furthermore, *A. baumannii* lacks the fast, generalized uptake of substances by non-specific porins such as OmpF, resulting in an overall membrane permeability of 5% relative to *E. coli*. The clear biologic plausibility of porin expression affecting antimicrobial resistance to selected hydrophilic antimicrobials such as carbapenems, and strong corollaries in other genera (i.e., conferral of carbapenem resistance in OprD-deficient *Pseudomonas aeruginosa*) have led to investigations into the role of porins in multidrug resistance in *A. baumannii* (Siroy et al., 2005; Rumbo et al., 2013; Smani and Pachón, 2013). Support for a role of porins in mediating polymyxin E resistance is limited, but not unprecedented (Pilonieta et al., 2009).

Transcriptomic data that compared AB307-0294 (wt) and its BfmR-deficient derivative AB307.70 (Δ*bfmR*) were consistent with *oprB* (ABBFA_00614, accession file CP001172.2) and *mapA* (ABBFA_00266, accession file CP001172.2), that encode the porins OprB and aquaporin, respectively, being regulated by BfmR. OprB is a glucose-sensitive porin homolog of OprB in *Pseudomonas aeruginosa* (Wylie et al., 1993). No information is available about the role of OprB in antimicrobial resistance in *A. baumannii*. Aquaporin (Rumbo et al., 2014) has previously been investigated as a carbapenem transport porin (Clark, 1996). Therefore, the role of these porins in modulating susceptibility to meropenem was studied in the XDR strain HUMC1; polymyxin E susceptibility was also assessed, although due to its known mechanism of action with LPS, an effect was predicted to be less likely.

BfmR has been previously shown to be essential for biofilm formation in non-XDR strains of *A. baumannii* (Thompson et al., 2012), and the biofilm state of bacteria has been associated with marked increases in antimicrobial resistance (Mah and O’Toole, 2001; Gurung et al., 2013; Macià et al., 2014). Therefore the hypothesis that BfmR mediated biofilm production contributed to the phenotype of carbapenem and polymyxin resistance in the XDR strain HUMC1 was also assessed.

## Materials and Methods

The transcriptome analysis of AB307-0294 and AB307.70 (Δ*bfmR*) when grown to log phase in minimal medium (MM) has been included as Supplementary Table 1. All other datasets generated during the current study are available from the corresponding author on reasonable request.

### Bacterial Strains and Media

*A. baumannii* strain HUMC1 (blood and lung isolate; sequence type 2, *bla* OXA-51-like, *bla* OXA-23-like, *bla* PER, *bla* GES, *aac*, IS *Aba1*) (El-Shazly et al., 2015) has a K4 capsular serotype (Wang-Lin et al., 2017) and was isolated from a patient in Los Angeles, CA (accession number NZ_LQRQ01000007.1). HUMC1Δ*bfmR*, HUMC1Δ*oprB*, and HUMC1Δ*mapA* are isogenic derivatives of HUMC1 generated by allelic exchange as described previously (Russo et al., 2009; Tucker et al., 2014) with the following selection marker and electroporation condition modifications as required for this XDR strain. For the generation of electrocompetent cells HUMC1 was grown overnight at 37°C in 5 mL of lysogeny broth (LB) (tryptone 10 g/L, yeast extract 5 g/L, NaCl 10 g/L-Becton Dickinson), shaking at 120 rpm, sub-cultured into 250 mL LB broth to a starting OD_600_ of 0.05 and harvested at OD_600_ of 0.6. The cells were washed 3x with ice-cold 10% glycerol and concentrated to 500 μL. HUMC1/pAT04 (a recombinase-containing plasmid that facilitated allelic exchange) was generated by transforming 100 μL of electrocompetent HUMC1 with 1.3 μg of pAT04 in 5 μL. Electroporation conditions were 1.8 kV, 1 mm cuvette, 100 Ω, 25 μF (Bio-Rad Gene Pulser). For the generation of isogenic mutants, 5 μg of a linear DNA fragment containing a hygromycin resistance gene conferring resistance at concentrations of 500 μg/mL flanked by the first and last 126 bp of the gene to be disrupted was utilized for allelic exchange (Primers listed in Supplementary Table 3) and transformed into electrocompetent HUMC1/pAT04. Recombinants were selected on LB plates (tryptone 10 g/L, yeast extract 5 g/L, NaCl 10 g/L, agar 15 g/L-Becton Dickinson) containing high concentration (500 μg/mL) of hygromycin, enabling selection of transformants in a strain resistant to aminoglycosides at physiologic concentrations (Luna et al., 2017). Successful gene disruption was confirmed by sequencing of PCR generated amplicons using primers outside of the gene in question. qRT-PCR was performed and confirmed a lack of transcript for the disrupted gene (Supplementary Figure 1, primers listed in Supplementary Table 3). A derivative cured of pAT04 was used for subsequent studies. Strains were maintained at -80°C in 50% glycerol-50% LB.

The procedures for obtaining human ascites fluid and human serum were reviewed and approved by the Western New York Veterans Administration Institutional Review Board; informed consent for ascites fluid was waived because it was collected from de-identified patients who were undergoing therapeutic paracentesis for symptoms due to abdominal distension. These individuals were not being treated with antimicrobials and were not infected with human immunodeficiency virus, hepatitis B virus, or hepatitis C virus. The ascites fluid was cultured to confirm sterility, divided into aliquots, and stored at -80°C. BBL^TM^ Mueller Hinton II cation adjusted (CAMH) broth consisted of 22 grams of powder per liter (Becton-Dickinson). MM consisted of 200 mL of solution A [2.0 g (NH4)_2_SO_4_, 6.0 g Na_2_HPO_4_, 3.0 g KH_2_PO_4_, 3.0 g NaCl, 0.011 g Na_2_SO_4_], 800 mL of solution B [0.2 g MgCl_2_, 0.0132 g CaCl_2_-2H_2_O, 0.0005 g FeCl_3_-7H_2_O, 2.9241 g citrate (trisodium salt dehydrate)], and 3 grams of casamino acids. HUMC1/pAT04 was grown in the presence of 80 μg/mL tetracycline, and HUMC1Δ*bfmR*, HUMC1Δ*oprB* and HUMC1Δ*mapA* were grown in the presence of 500 μg/mL of hygromycin.

### Transcriptome Analysis (RNA-Seq)

Transcriptome analysis (RNA-Seq) was used to identify potential BfmR-regulated mediators of antibiotic resistance in the clinical *A. baumannii* strain of AB307-0294. RNA sequencing was performed at the University at Buffalo-SUNY Genomics and Bioinformatics Core Facility. Illumina TruSeq RNA sample preparation kit was used to prepare cDNA libraries from RNA samples from AB307-0294 and AB307.70 (Δ*bfmR*) grown to log phase in MM. Ribosome-depleted RNA samples were cleaved into fragments, the first strand was reverse transcribed to cDNA (Invitrogen SuperScript II reverse transcriptase), and then the second strand was synthesized (Illumina Second Strand Master Mix). After end repair and ligation, the products were enriched and purified to create a cDNA library for both AB307-0294 and AB307.70. The libraries were then quantified using Invitrogen Picogreen assay and Kapa Biosystems library quantification kit. Fragment Analyzer High Sensitivity NGS kit (Advance Analyticals) was used to confirm the quality and size of the cDNA libraries. The cDNA libraries were then normalized, pooled, and sequenced (Illumina HiSeq 2500). Transcriptome analysis was also performed at the University at Buffalo Core Genomics facility, using the TopHat Cufflinks pipeline. The data was aligned against the reference accession NC_011595.1^1^. The dispersion (variance) was determined at the gene-by-gene level and significantly changing genes were determined. Next, the identity of each significantly affected gene, if previously studied, was determined. The function of genes not previously studied was postulated by BLAST search^2^ for homologous sequences and by protein functional analysis. *oprB* and *mapA*, which were significantly regulated by BfmR and had plausible mechanisms for contributing to antimicrobial resistance, were chosen for further study. The complete results of the transcriptome and Clusters of Orthologous Groups (COG) analyses are presented in Supplementary Tables 1, 2; (the ‘old’ gene locus designations were used for COG analysis). Since these data were produced, file NC_011595.1 has been replaced by revised CP001172.2. Locus tags therefore no longer link directly to the updated accession https://www.ncbi.nlm.nih.gov/nuccore/CP001172. Transcriptome data was confirmed by qPCR according to Minimal Information for Publication of Quantitative Real-Team PCR Experiments (MIQE) guidelines. The qPCR reference gene was ABBFA_RS00075 (accession file NC_011595.1), which encodes the 16S ribosomal RNA gene.

### Growth in LB, MM, and Human Ascites Fluid

Growth experiments in LB, MM, and human ascites were performed as previously described (Russo et al., 2016). MM was used since this medium more closely mimics growth conditions within the human host where nutrients are limited compared to rich laboratory medium. Ascites was used because it is both nutrient limited, contains host defense factors such as complement and antimicrobial peptides, and larger quantities are more readily available compared to human serum.

### Antimicrobial Susceptibility Testing

Assays were performed in 96-well microtiter plates. Wells contained final drug concentrations from 0 to 128 μg/mL of meropenem (AstraZeneca Pharmaceuticals LP) and 0–6 μg/mL of polymyxin E (colistin Sulfate Salt, Sigma C4461). HUMC1 and isogenic derivatives were grown in CAMH broth at 37°C overnight and diluted in CAMH to 1-2 × 10^5^ CFU per well (CLSI, 2018). Control wells contained growth medium alone. Microtiter plates were incubated for 18 h at 37°C. Bacterial susceptibilities to antibiotics was quantitated by the optical density at 600 nm (OD_600_) of each well was measured in a microplate spectrophotometer (SpectraMax 190; Molecular Devices) at 26°C and/or bacterial CFU were enumerated by serial 10-fold dilutions. The minimal bactericidal concentration (MBC) was defined as the lowest antimicrobial concentration that resulted in <10^1^ CFU/mL. At least 2 independent experiments, with cumulative repetitions of 4–16, were performed for each strain and antimicrobial concentration. Comparisons between HUMC1 and its isogenic mutant derivatives HUMC1 Δ*bfmR*, HUMC1Δ*mapA* or HUMC1Δ*oprB* when tested against meropenem or polymyxin E were performed in parallel to control for inter-test variability.

### Biofilm Assay

Assays were performed in 96-well microtiter plates as described (O’Toole, 2011). In brief, 100 μL of CAMH containing 1 × 10^7^CFU/mL of the strain to be tested was placed in each well. The plates were incubated at 37°C overnight without shaking. The next day, the medium containing planktonic cells was removed and the wells were washed gently 3 times by submerging the plate in distilled H_2_O. One hundred twenty-five μL of 0.1% crystal violet was then placed in each well. The plates were incubated at room temperature for 10 min, washed again 3 times with distilled H_2_O, blotted, and air dried. After drying, 125 μL of 30% acetic acid was placed in each well, and the plates were incubated for 10–15 min. The solution containing solubilized crystal violet was transferred to a new 96-well plate and biofilm was quantitated by measurement of absorbance at 550 nm in a microplate spectrophotometer (SpectraMax 190; Molecular Devices) at 26°C; which reflects biofilm formation. Wells not inoculated with bacteria served as the negative control.

### DNase-Modified Susceptibility Studies

Assays were performed in 96-well microtiter plates. HUMC1 and HUMC1Δ*bfmR* were grown overnight at 37°C in LB and on the next day diluted in CAMH broth so that each well contained 1-2 × 10^7^ CFU/mL in 100 μL. Control wells contained CAMH broth only. Wells contained final drug concentrations from 0 to 128 μg/mL of meropenem and 0–8 μg/mL of polymyxin E. DNase was used to decrease biofilm formation. DNase-containing wells were supplemented with a final concentration of 10 U/mL DNase in 2.5 mM MgCl_2_ and 0.1 mM CaCl_2_ (Thermo Fisher Scientific DNase I, RNase free); controls were supplemented with an equal volume of CAMH broth. The microtiter plate was incubated for 24 h at 37°C. The optical density at 600 nm (OD_600_) of each well was measured in a microplate spectrophotometer (SpectraMax 190; Molecular Devices) at 26°C. Bacterial CFU were enumerated by serial 10-fold dilutions. Biofilms were disrupted by gently pipetting up and down in each well prior to bacterial enumeration, thus ensuring that total bacterial growth, including both planktonic and biofilm state, was assessed in each measurement. At least 2 independent experiments, with total repetitions of 6–16, were performed for each strain and antimicrobial concentration.

### Statistics

Continuous data were assessed for normality and are presented as means ± standard errors of the means (SEM.) *P* values of ≦0.05 were considered statistically significant. To normalize *ex vivo* growth/survival data, log_10_-transformed values were utilized. The area under each curve was calculated, and these areas were compared using two-tailed unpaired t tests (Prism 8.2 for MacIntosh; GraphPad Software, Inc.). Statistical analysis was not considered reliable for the transcriptome analysis due a sample size of one for each strain.

## Results

### Loss of BfmR Increases Susceptibility to Meropenem and Polymyxin E in HUMC1 With Planktonic Growth

**FIGURE 1:**
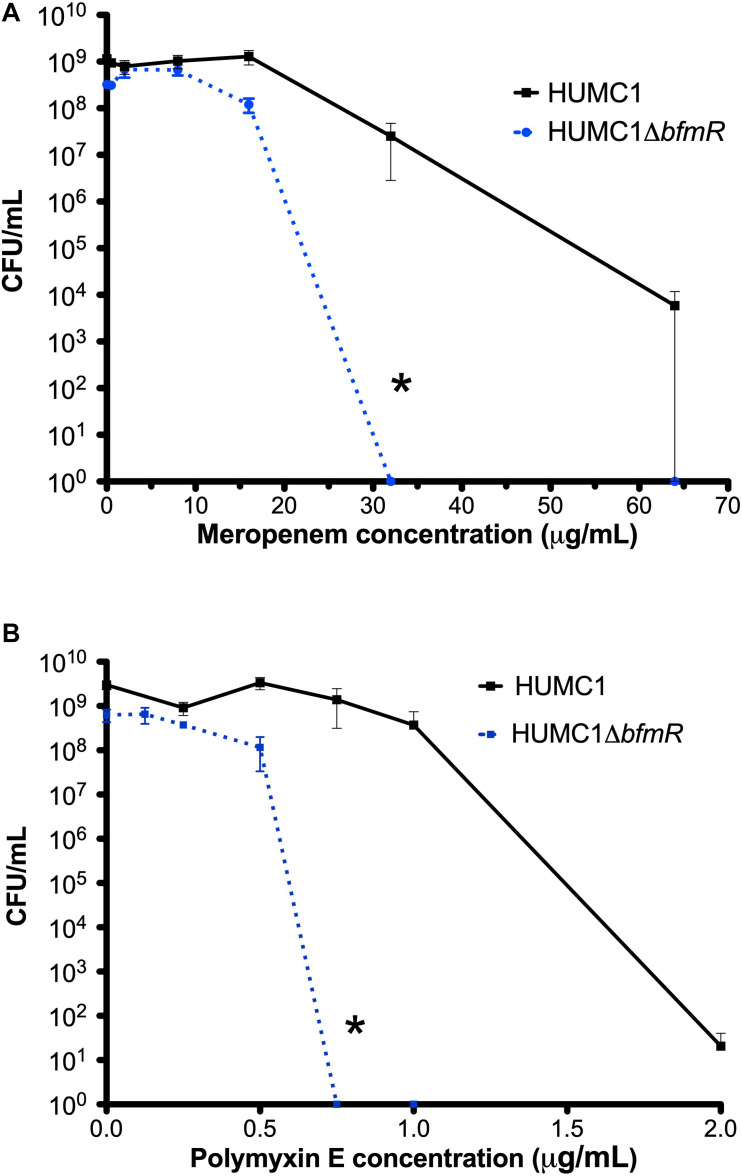
Comparison of the growth/survival of HUMC1 and HUMC1Δ*bfmR* when exposed to various concentrations of meropenem (0–64 μg/mL, panel A) and polymyxin E (0–2 μg/mL, panel B) after 18 h incubation in Mueller Hinton II cation-adjusted broth at 37°C. Data are means ± SEM, *n* = 4; *P < 0.001 and *P = 0.0094, respectively, with comparisons of log-transformed AUC by unpaired *t* test.

To confirm an effect of BfmR on increasing resistance to meropenem and polymyxin E (Russo et al., 2016; Geisinger et al., 2018) in the XDR *A. baumannii* strain HUMC1, the isogenic mutant construct HUMC1Δ*bfmR* was generated for comparison with its wild-type parent. HUMC1Δ*bfmR* demonstrated a significantly increased susceptibility to meropenem and polymyxin E when assessed in CAMH (Figure 1).

A prior transcriptome analysis of AB307-0294 (wt) and its BfmR deficient derivative AB307.70 (Δ*bfmR*) informed on gene products that could contribute to BfmR-regulated antimicrobial resistance to meropenem and polymyxin E in HUMC1.

**FIGURE 2:**
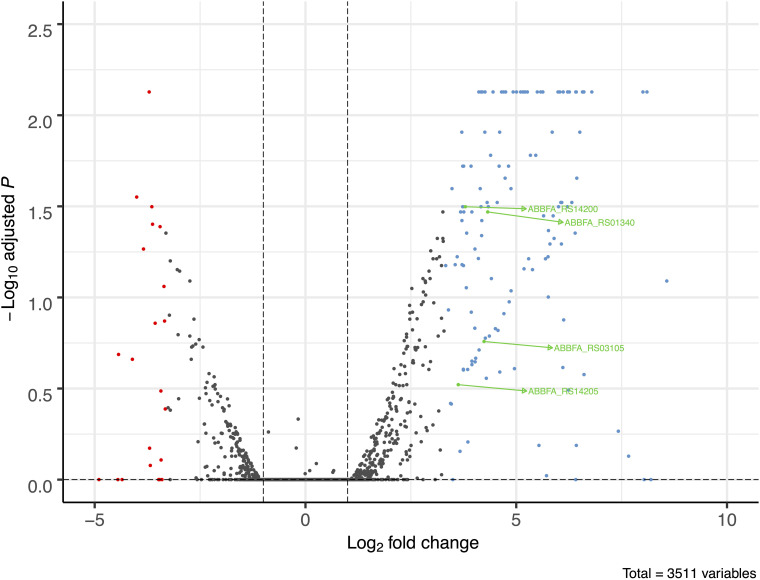
Volcano plot of transcriptome analysis of AB307-0294 and its BfmR-deficient derivative AB307.70 (Δ*bfmR*). The ratio of RNA abundance in AB307-0294/AB307.70 (Δ*bfmR*) was calculated and plotted as log2-fold change. The dashed vertical lines left of zero marks a log2- fold change of -1 and the dashed line right of zero shows marks a log2 fold-change greater than 1, corresponding to a fold change of 0.5 and 2, respectively. A total of 3511 genes were assessed, those upregulated >10-fold are marked in blue, those downregulated >10-fold are marked in red, and those of interest for this study are marked in green, ABBFA_RS14200 (*bfmS*), ABBFA_RS01340 (*mapA*), ABBFA_RS03105 (*oprB*), and ABBFA_RS14205 (*bfmR*). The plot was generated using R package version 1.4.0, https://github.com/kevinblighe/EnhancedVolcano.

**FIGURE 3:**
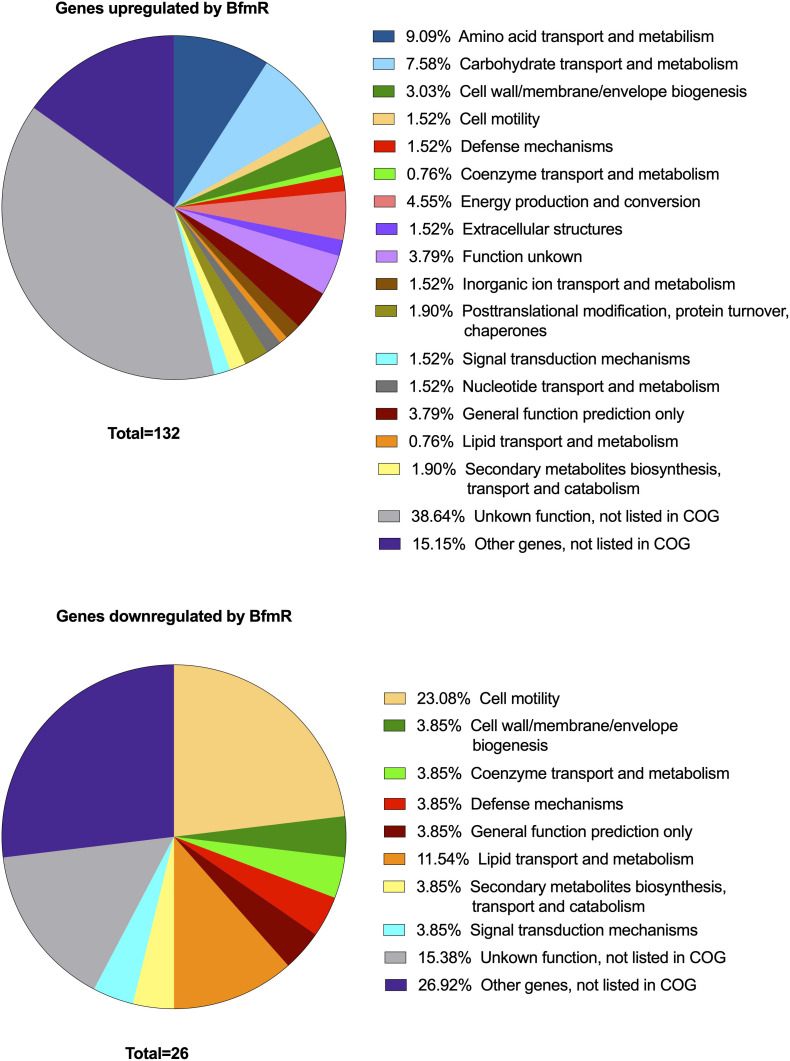
Cluster of orthologous groups (COG) analysis of genes >10-fold up- (*N* = 132) or down- (*N* = 26) regulated by BfmR identified in the transcriptome analysis of AB307-0294 and its BfmR-deficient derivative AB307.70 (Δ*bfmR*). *Integrated Microbial Genomes and Microbiomes* (img.jgi.doe.gov) COG listings for AB307-0294 were used to identify categories for the differentially regulated genes using locus tags (e.g., ABBFA_xxxxxx).

A total of 158 genes (4.5% of 3511 genes present in AB307-0294) were identified in which the RNA expression (abundance) was increased or decreased by >10-fold in AB307-0294 compared to AB307.70 (Δ*bfmR*) when grown to log phase in MM (Figure 2). This cohort of genes was predicted to be positively (*n* = 132) or negatively (*n* = 26) regulated (directly or indirectly) by BfmR (Figure 2, Supplementary Table 1, marked in blue (upregulated) or red (downregulated). A cluster of orthologous groups (COG) analysis of these 158 genes and their putative function is depicted in Figure 3 and Supplementary Table 2. For the COG analysis the differentially regulated genes were searched against the *Integrated Microbial Genomes and Microbiomes (img.jgi.doe.gov)* COG listings for AB307-0294 using the ‘old’ gene locus designations (ABBFA_xxxxxx, Supplementary Tables 1, 2). The COG categories of amino acid (9.09%) and carbohydrate (7.58%) transport and metabolism contained the largest proportion of BfmR positively regulated genes, whereas cell motility (23.08%) contained the largest proportion of negatively regulated genes. However, of note, 38.64% and 15.15% of upregulated genes were not listed in the COG database and were categorized by us as having unknown function (hypothetical proteins) and other genes (i.e., not listed in COG) respectively. Likewise, 15.38% and 26.92% of downregulated genes were categorized as having unknown function and other genes, respectively. The specific genes in each COG category are delineated in Supplementary Table 2.

Of note, the transcriptomic analysis is consistent with BfmR positively regulating its synthetic operon *bfmSR*. The expression of *bfmS* and *bfmR* was increased by 13.86-fold and 12.33-fold, respectively, in AB307-0294 compared to AB307.70 (Δ*bfmR*).

A transcriptome analysis of AB307-0294 and AB307.70 (Δ*bfmR*) identified OprB and aquaporin as potential mediators of BfmR-regulated antimicrobial resistance in HUMC1.

Two of the genes identified, *oprB*, (encodes the porin OprB) and *mapA* [encodes aquaporin (Omp33-36)] were hypothesized to contribute to antimicrobial resistance (Supplementary Table 1, marked in green). The RNA expression of *oprB* and *mapA* was increased by 18.8-fold and 20-fold, respectively, in AB307-0294 compared to AB307.70 (Δ*bfmR*) (*P* = 0.0065 and *P* = 0.0005, respectively). This result was confirmed by qPCR; *oprB* and *mapA* expression was increased by 10.4-fold and 25.6-fold respectively in AB307-0294 compared to AB307.70 (Δ*bfmR*).

Next, RNA expression was studied in HUMC1 and HUMC1Δ*bfmR* by qPCR when grown to log phase in MM. *oprB* and *mapA* expression was increased by 9.8-fold and 8.5-fold, respectively in HUMC1 compared to HUMC1Δ*bfmR* (*P* = 0.01 and *P* = 0.02, respectively). Lastly, RNA expression was studied in HUMC1 and HUMC1Δ*bfmR* by qPCR when grown to log phase in CAMH since growth media can affect gene expression, and this medium is the standard used for susceptibility testing. *oprB* and *mapA* expression was increased by 7.5 ± 0.52-fold and 6.4 ± 0.80-fold, respectively in HUMC1 compared to HUMC1Δ*bfmR* (*N* = 3, *P* = 0.0002, and *P* = 0.0026, respectively).

### Loss of the Porin OprB Does Not Affect Meropenem or Polymyxin E Resistance in HUMC1

**FIGURE 4:**
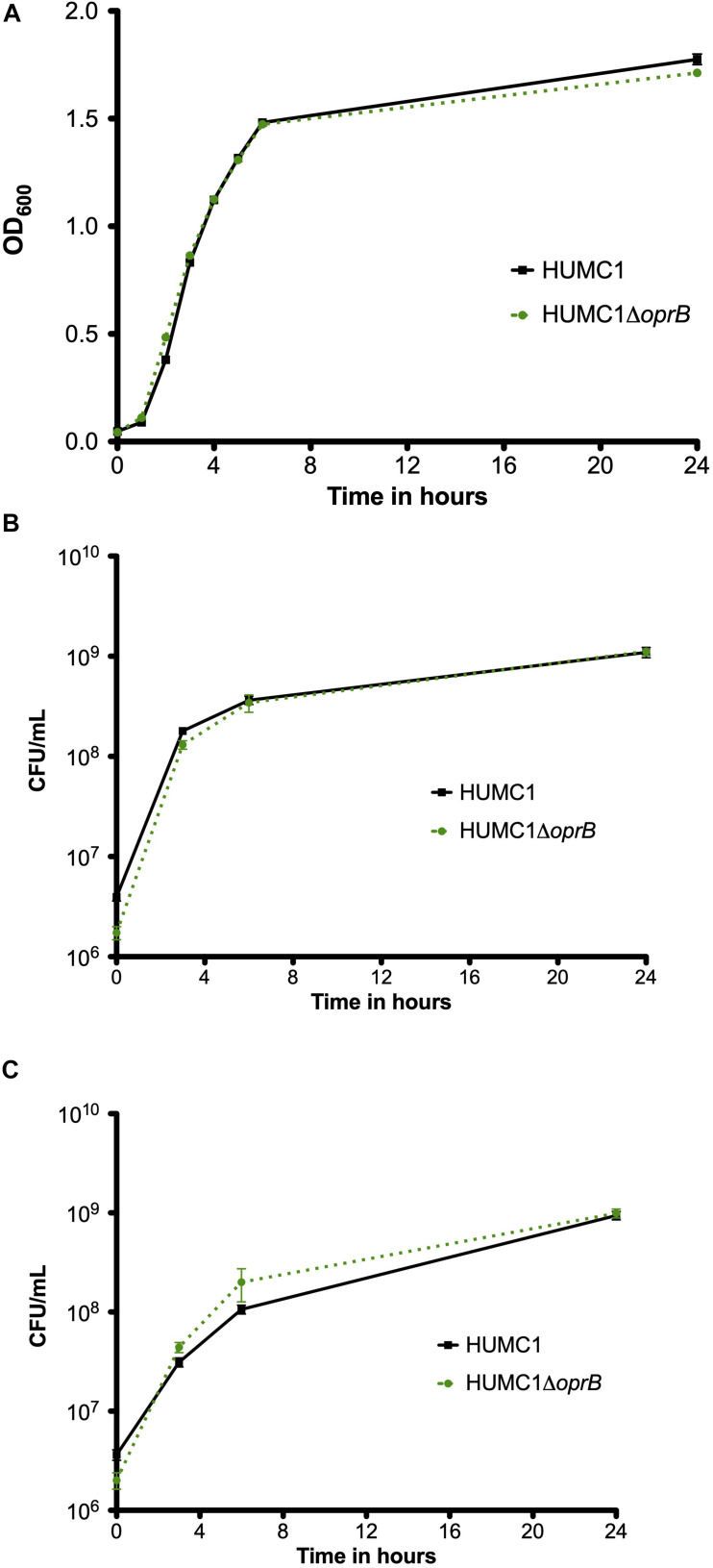
Growth comparison of HUMC1 and HUMC1Δ*oprB* in lysogeny broth (panel **A**), minimal medium (panel **B**), and human ascites fluid (panel **C**). Data are means ± SEM, *n* = 6, *P* = 0.1661, *P* = 0.687, and *P* = 0.4545, respectively, with comparisons by unpaired *t* test of log-transformed AUC for experiments measuring CFU/mL and of AUC for experiments measuring optical density.

To assess whether OprB contributes to antimicrobial resistance in HUMC1, the isogenic mutant construct HUMC1Δ*oprB* was generated for comparison with its wild-type parent. First, growth of HUMC1 and HUMC1Δ*oprB* was assessed in LB, MM, and human ascites. No significant differences were observed (Figure 4), demonstrating that HUMC1Δ*oprB* does not possess a generalized growth defect in laboratory or clinically relevant media.

**FIGURE 5:**
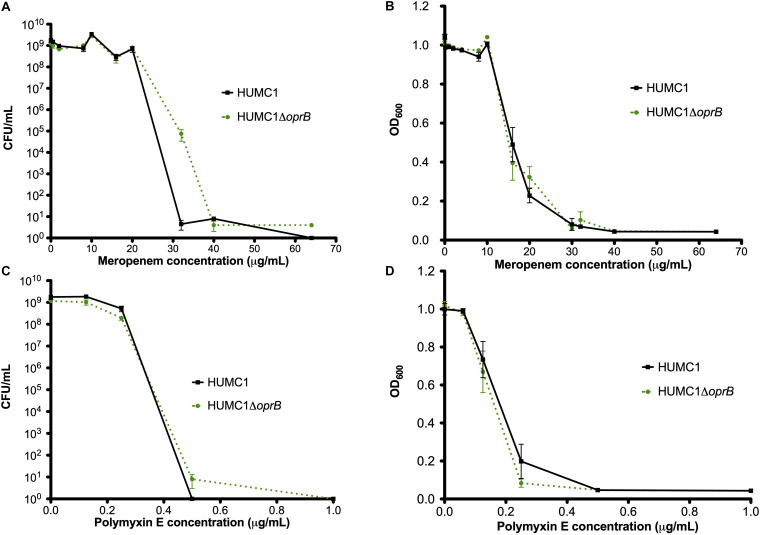
Comparison of the growth/survival of HUMC1 and HUMC1Δ*oprB* in in Mueller Hinton II cation-adjusted medium when exposed to various concentrations of meropenem (0–64 μg/mL, panels **(A)** [CFU/mL] and **(B)** [optical density at 600 nm]) and polymyxin E (0–1.5 μg/mL, panels **(C)** [CFU/mL] and **(D)** [optical density]) after 18 h incubation at 37°C. Data are means ± SEM, *n* = 3 for experiments measuring CFU and *n* = 12 for experiments measuring optical density. *P* = 0.0625, *P* = 0.5995, *P* = 0.7396, and *P* = 0.2092, respectively, with comparisons by unpaired *t* test of log-transformed AUC for experiments measuring CFU/mL and of AUC for experiments measuring optical density.

Next, the susceptibilities of HUMC1 and HUMC1Δ*oprB* to meropenem and polymyxin E were assessed both by measurement of optical density and quantification of CFU/mL at relevant meropenem and polymyxin E concentrations. The effect of meropenem and polymyxin E on HUMC1 and HUMC1Δ*oprB* was similar (Figure 5).

### Loss of Aquaporin Does Not Affect Meropenem or Polymyxin E Resistance in HUMC1

**FIGURE 6:**
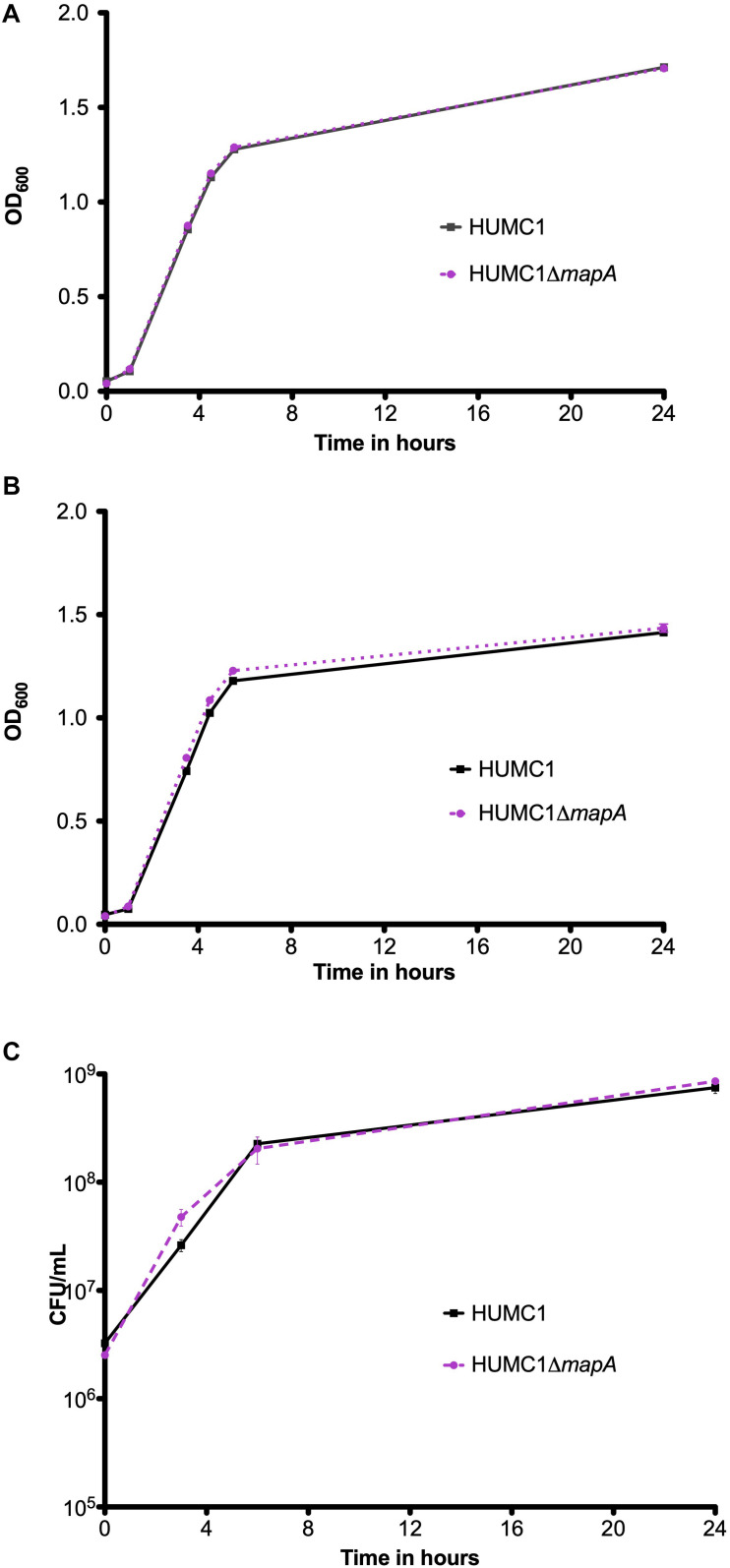
Growth comparison of HUMC1 and HUMC1Δ*mapA* in lysogeny broth (panel **A**), minimal medium (panel **B**), and human ascites fluid (panel **C**). Data are means ± SEM, *n* = 3, *P* = 0.7055, *P* = 0.0699, and *P* = 0.8647, respectively, with comparisons by unpaired *t* test of log-transformed AUC for experiments measuring CFU/mL and of AUC for experiments measuring optical density.

The isogenic mutant construct HUMC1Δ*mapA* was generated for comparison with its wild-type parent. Then, growth of HUMC1 and HUMC1Δ*mapA* were assessed in LB, MM, and human ascites. No significant differences were observed (Figure 6), demonstrating that HUMC1Δ*mapA* does not possess a generalized growth defect in laboratory or clinically relevant media.

**FIGURE 7:**
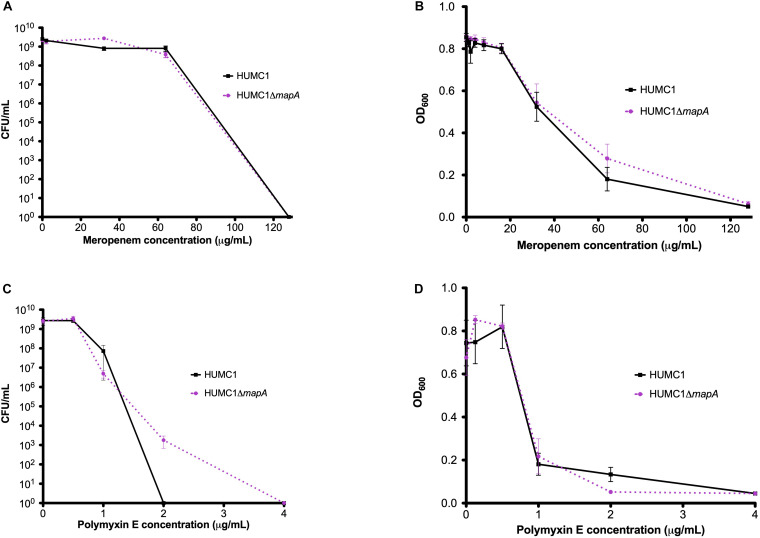
Comparison of the growth/survival of HUMC1 and HUMC1Δ*mapA* in Mueller Hinton II cation-adjusted broth in various concentrations of meropenem [0–128 μg/mL, panels **(A)** (CFU/mL) and **(B)** (optical density)] and polymyxin E [0–4.0 μg/mL, panels **(C)** (CFU/mL) and **(D)** (optical density)] after 18 h incubation at 37°C. Data are means ± SEM, *n* = 3–5 for experiments measuring CFU and *n* = 8 for experiments measuring optical density. *P* = 0.8539, *P* = 0.4220, *P* = 0.6627, and *P* = 0.9916, respectively, with comparisons of log-transformed AUC by unpaired *t* test for experiments measuring CFU/mL and comparisons of AUC by unpaired *t* test for experiments measuring optical density.

Susceptibilities of HUMC1 and HUMC1Δ*mapA* to meropenem and polymyxin E were then assessed by measuring optical density and by quantification of CFU/mL at relevant antimicrobial concentrations. The effect of meropenem and polymyxin E on HUMC1 and HUMC1Δ*mapA* was similar (Figure 7). These data do not support a role for aquaporin in BfmR-mediated intrinsic antimicrobial resistance in HUMC1.

### BfmR Regulates Biofilm Production in HUMC1

**FIGURE 8:**
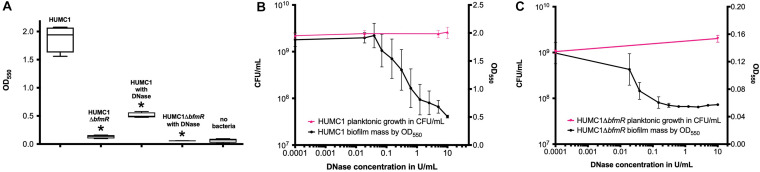
Panel **(A)** Optical density at 550 nm after crystal violet biofilm staining for HUMC1 and HUMC1Δ*bfmR* with and without 10 U/mL DNase after incubation in Mueller Hinton II cation-adjusted broth for 24 h at 37°C. Values are means, boxes the 25th and 75th percentile, whiskers minimum and maximum. HUMC1 mean absorbance = 1.88 ± 0.12; HUMC1 with DNase mean absorbance = 0.51 ± 0.02, *P* < 0.0001). HUMC1Δ*bfmR* mean absorbance = 0.1325 ± 0.01564; HUMC1Δ*bfmR* with DNase mean absorbance = 0.0575 ± 0.001323, *P* = 0.0031). Data are means ± SEM, with comparisons by unpaired *t* test, *n* = 4. Panel **(B)** Optical density at 550 nm after crystal violet biofilm staining for HUMC1 at DNase concentrations 0–10 U/mL, with corresponding growth of planktonic bacteria in CFU/mL. Panel **(C)** Optical density at 550 nm after crystal violet biofilm staining for HUMC1Δ*bfmR* at DNase concentrations 0–10 U/mL, with corresponding growth of planktonic bacteria in CFU/mL.

HUMC1-mediated biofilm production was greater in CAMH broth compared to MM (mean absorbance at 550 nm 1.88 ± 0.12 and 0.2802 ± 0.0100, respectively). As expected, the loss of BfmR decreased biofilm formation, which was quantitatively greater in CAMH broth than in MM. A significant 92.9% reduction was observed in CAMH broth (mean absorbance at 550 nm 1.88 ± 0.12 for HUMC1 and 0.1325 ± 0.016 for HUMC1Δ*bfmR*; *n* = 4, *p* < 0.0001) (Figure 8A), whereas a significant 43.4% reduction in biofilm production was observed in MM without casamino acids (mean absorbance at 550 nm 0.2802 ± 0.0100 for HUMC1 and 0.1954 ± 0.0051 for HUMC1Δ*bfmR*; *n* = 24, *p* < 0.0001) (data not shown).

### In HUMC1, BfmR Mediated Resistance to Polymyxin E Can Be Partially Attributed to Its Ability to Regulate Both Biofilm Formation and Factors Additional to Biofilm Formation

**FIGURE 9:**
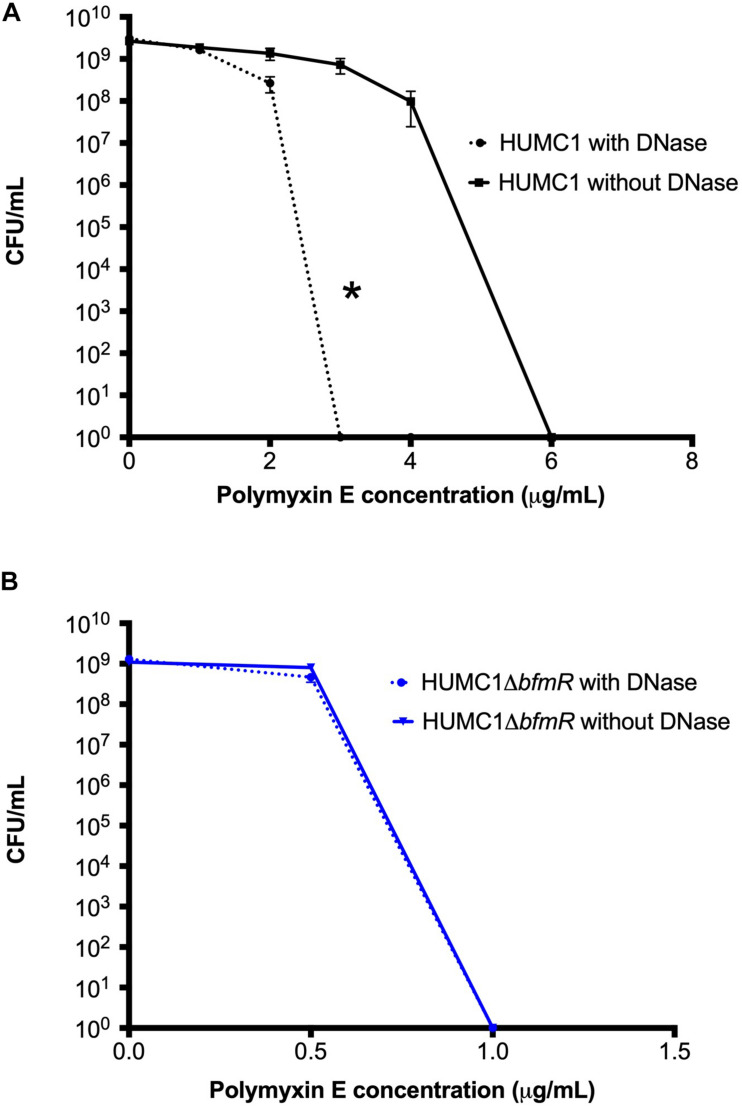
Panel **A:** Comparison of the growth/survival of HUMC1 in Mueller Hinton II cation-adjusted broth with and without 10 U/mL DNase in various concentrations of polymyxin E (0–8 μg/mL) after 24 h incubation at 37°C. Data are means ± SEM, *n* = 12, *P* = 0.0134, with comparisons of log-transformed AUC by unpaired *t* test. Panel **(B)** Comparison of the growth/survival of HUMC1 Δ*bfmR* in Mueller Hinton II cation-adjusted broth with and without 10 U/mL DNase in various concentrations of polymyxin E (0–1.5 μg/mL) after 24 h incubation at 37°C. Data are means ± SEM, *n* = 6, *P* = 0.1248, with comparisons of log-transformed AUC by unpaired *t* test.

Next, the bactericidal activity of polymyxin E against HUMC1 and HUMC1Δ*bfmR* grown under biofilm inducing conditions was assessed. Not surprisingly, HUMC1 grown under biofilm inducing conditions demonstrated increased resistance to polymyxin E (MBC 6 μg/mL, Figure 9) compared to planktonic growth conditions (MBC 2 μg/mL, Figure 1B). To gain insight into the contribution of BfmR regulated biofilm production to the observed increased resistance to polymyxin E under biofilm inducing conditions, HUMC1Δ*bfmR* grown under biofilm inducing conditions was exposed to varying concentrations of polymyxin E. The 92.9% reduction in biofilm formation mediated by loss of BfmR (Figure 8A) was associated with an increased sensitivity to polymyxin E (MBC 1 μg/mL, Figure 9). However, since BfmR has pleiotropic effects, one cannot conclude that the increased sensitivity observed with the loss of BfmR was solely due to decreased biofilm formation.

Therefore, to delineate the relative role of biofilm formation on the susceptibility to polymyxin E, a survey of various assay conditions was performed to identify the reagents and conditions that provided optimal inhibition of biofilm formation without affecting bacterial growth (Figures 8B,C). In CAMH broth, after incubation for 24 h without shaking, DNase (10 U/mL) caused a significant 73% reduction in HUMC1 mediated biofilm formation (mean absorbance at 550 nm 1.88 ± 0.12 for HUMC1 and 0.51 ± 0.02 for HUMC1 with DNase; *n* = 4, *p* < 0.0001) (Figure 8A). There was no effect on total bacterial growth under these conditions (Figures 8B,C), therefore studies were performed under these conditions.

Then, HUMC1 was grown under biofilm inducing conditions in the presence of DNase (10 U/mL) and varying concentrations of polymyxin E. The 73% DNase-mediated reduction in biofilm formation produced by HUMC1 resulted in a decrease in the MBC to polymyxin E from 6 μg/mL (in the absence of DNase) to 3 μg/mL (in the presence of DNase) (Figure 9A). These data demonstrate that HUMC1 mediated biofilm formation decreases susceptibility to polymyxin E. However, the small 4% reduction in biofilm formation when HUMC1Δ*bfmR* was grown under biofilm inducing conditions in the absence and presence of DNase (residual biofilm produced by HUMC1Δ*bfmR* decreased from 7.1% (in the absence of DNase) to 3% (in the presence of DNase) did not further increase sensitivity to polymyxin E (MBC 1 μg/mL for both conditions, Figure 9B), suggesting this small change in biofilm formation has no effect on polymyxin E susceptibility.

### The BfmR Mediated Resistance of HUMC1 to Meropenem Is More Likely Due to BfmR Regulated Factors That Do Not Affect Biofilm Formation

**FIGURE 10:**
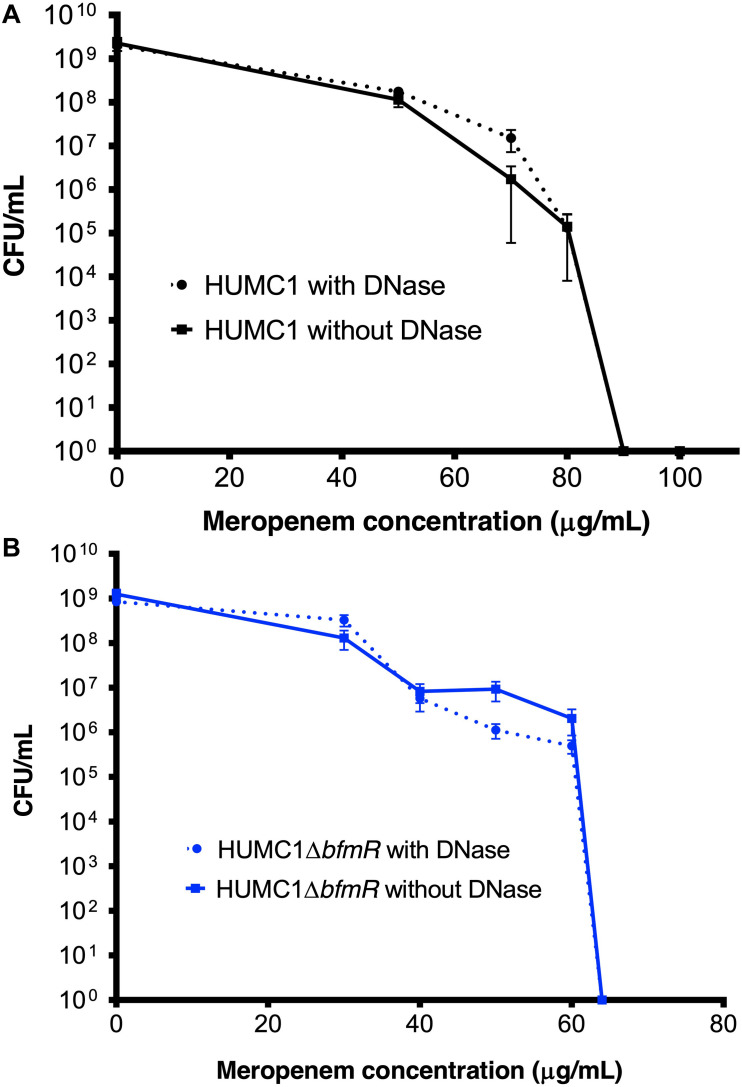
**(A)** Comparison of the growth/survival of HUMC1 with and without 10 U/mL DNase in Mueller Hinton II cation-adjusted broth in various concentrations of meropenem (0–100 μg/mL) after 24 h incubation at 37°C. **(B)** Comparison of the growth/survival of HUMC1Δ*bfmR* with and without 10 U/mL DNase in Mueller Hinton II cation-adjusted broth in various concentrations of meropenem (0–60 μg/mL). Data are means ± SEM, *n* = 6, *P* = 0.3669, and *P* = 0.9343, respectively, with comparisons of log-transformed AUC by unpaired *t* test.

Lastly, the bactericidal activity of meropenem against HUMC1 and HUMC1Δ*bfmR* grown under biofilm inducing conditions was assessed. HUMC1 grown under biofilm inducing conditions demonstrated a modest increased resistance to meropenem, best compared at the meropenem concentrations that result in a reduction from approximately 1 × 10^9^ CFU to 1 × 10^4^ CFU (85 μg/mL under biofilm inducing conditions (Figure 10A) compared to 64 μg/mL (Figure 1A) under planktonic growth conditions). To gain insight into the contribution of BfmR regulated biofilm production to the observed modest increase in resistance to meropenem under biofilm inducing conditions HUMC1Δ*bfmR* grown under biofilm inducing conditions was exposed to varying concentrations of meropenem. The 92.9% reduction in biofilm formation mediated by loss of BfmR (Figure 8A) was associated with an increased sensitivity to meropenem (MBC 64 μg/mL, Figure 10B). However, since BfmR has pleiotropic effects, one cannot conclude that the increased sensitivity observed with the loss of BfmR was solely due to decreased biofilm formation.

Therefore, to delineate the relative role of biofilm formation on the susceptibility to meropenem, HUMC1 was grown under biofilm inducing conditions in the presence of DNase (10 U/mL) and varying concentrations of meropenem. The 73% DNase-mediated reduction in biofilm formation produced by HUMC1 had no effect on the sensitivity to meropenem (MBC 90 μg/mL in the presence and absence of DNase (Figure 10A). In addition, the 4% reduction in biofilm formation when HUMC1Δ*bfmR* was grown under biofilm inducing conditions in the presence of DNase (residual biofilm produced by HUMC1Δ*bfmR* decreased from 7.1% (in the absence of DNase) to 3% (in the presence of DNase), albeit very small, also had no effect on sensitivity meropenem (MBC 64 μg/mL for both conditions, Figure 10B).

## Discussion

*Acinetobacter baumannii* is one of the most problematic XDR Gram-negative pathogens due to the combination of its intrinsic and acquired antimicrobial resistance. We have previously demonstrated that the transcriptional regulator BfmR enhances intrinsic resistance to meropenem and polymyxin E in antimicrobial sensitive clinical strains (Russo et al., 2016). In this report we assessed this phenotype in the clinically relevant XDR strain HUMC1 and assessed potential mechanisms by which this occurred. First, we confirmed that the loss of BfmR increases susceptibility to meropenem and polymyxin E in HUMC1. Next, to identify candidate factors that contributed to BfmR regulated meropenem and polymyxin E resistance, we utilized previously generated transcriptomic data that compared the antimicrobial sensitive clinical isolate AB307-0294 (wt) and its BfmR-deficient derivative AB307.70 (Δ*bfmR*). These data identified the BfmR regulated genes *oprB* and *mapA*, which encode the porins OprB and aquaporin, respectively. Then, we confirmed that these genes were upregulated by BfmR in the XDR strain HUMC1. However, surprisingly, despite reports from others (Bou et al., 2000; Rumbo et al., 2014) and biologic plausibility of porins as contributors to BfmR’s mediated antimicrobial resistance phenotype against hydrophilic antimicrobials such as meropenem (Vila et al., 2007; Papp-Wallace et al., 2011), our work has found no significant effect of OprB or aquaporin on meropenem susceptibilities in HUMC1 (Figures 5, 7). Due to its known interaction with LPS, it was deemed unlikely that these porins were responsible for BfmR’s mediated resistance to polymyxin E and this proved to be the case. Next, we assessed whether the BfmR-regulated increase in biofilm formation was contributory. Surprisingly, biofilm formation increased resistance to polymyxin E, but had little, if any effect, on meropenem activity (Figures 9, 10). The BfmR mediated resistance of HUMC1 to meropenem and perhaps to polymyxin E in part, was due to BfmR regulated factors that do not affect biofilm formation. These findings increase our understanding on the mechanisms by which BfmR mediates intrinsic antimicrobial resistance; however, other yet unidentified factors are also operative.

The literature to date on the role of *A. baumannii* porins in antimicrobial (primarily carbapenem) resistance has been fraught with contradictions and uncertainties, likely related to the complexities and variety of mechanisms by which *A. baumannii* acquires antimicrobial resistance, as well as to the substantial genetic and phenotypic differences between laboratory strains and clinical isolates of *A. baumannii*. *A. baumannii* genomes are highly heterologous. They are capable of significant genetic plasticity, by means of genetic islands, single nucleotide polymorphisms, and mobile genetic elements (Liu et al., 2014). These genetic variations have practical implications in the study of virulence and drug resistance in *A. baumannii*, as findings in laboratory strains do not necessarily correlate with findings in clinical isolates. Previous studies on mechanisms of antimicrobial resistance in *A. baumannii* have noted marked differences in profiles of outer membrane protein expression in clinical, drug-resistant strains compared to the ATCC 17978 reference strain (Lu et al., 2009). Tight association of OXA carbapenemases with outer membrane proteins occurs in drug-resistant clinical isolates, but not in the reference strain (Ambrosi et al., 2017). BfmR has been shown to have differing effects on capsule production in a clinical isolate compared with ATCC 17978 (Russo et al., 2016). Thus, our work has focused exclusively on clinical isolates to provide the data most translatable to patient care. Further, in this study we assessed the XDR strain HUMC1 since understanding antimicrobial resistance in this genomic background is particularly relevant for treatment challenges posed by such isolates.

The transcriptome analysis performed on AB307-0294 will serve as an important resource for future studies on genes regulated by BfmR. Previous work had established that in the ATCC strain AB19606 BfmR increased the expression of genes that encoded the Csu pili chaperone-usher system, which contributes to biofilm formation (Tomaras et al., 2008). Transcriptomic data from AB307-0294 supports this result: *csuAB* (gene 1221, ABBFA_RS06100, ABBFA_001232) increased 95.17-fold, *csuA* (gene1222, ABBFA_RS06105, ABBFA_001233) increased 11.01-fold, *csuC* (gene1224, ABBFA_RS06115, ABBFA_001235) increased 14.45-fold, and *csuD* (gene1225, ABBFA_RS06120, ABBFA_001236) increased 10.87-fold (Supplementary Tables 1, 2). The exact increase in the expression of *csuB* could not be calculated due to a lack of detection of its transcript in AB307.70. Likewise, the two genes (*oprB* and *mapA*) studied in this report were both up-regulated by BfmR in HUMC1. Although, the extent to which the transcriptomic analysis of AB307-0294 will prove to be broadly applicable remains unclear, these data can serve as a roadmap for identifying genes of interest, followed by confirmation in the strain being studied, such as was done for *oprB* and *mapA* in this report. Lastly, the transcriptome was assessed in planktonic bacteria. Previous work has shown that that the transcriptome differs when *A. baumannii* is in a biofilm compared to a planktonic state (Rumbo-Feal et al., 2013). Therefore, it is possible that BfmR-mediated gene regulation could differ between these states.

It remains unclear whether the effect of biofilm on polymyxin E susceptibility is primarily direct or indirect. In *Pseudomonas aeruginosa*, release of extracellular DNA chelates cations, which induces the cationic antimicrobial peptide resistance operon *pmrCAB* (Mulcahy et al., 2008). This operon confers resistance to cationic antimicrobials (such as polymyxin E), but not to beta lactams, by means of lipid A modifications. Interestingly, *A. baumannii* also possesses the *pmrCAB* operon, which has been implicated in polymyxin E resistance (Adams et al., 2009) and heteroresistance (Charretier et al., 2018). However, it has not been specifically established that extracellular DNA induces this operon in *A. baumannii*. If this mechanism is operative in *A. baumannii*, the role of BfmR in polymyxin E resistance may be indirect, by its stimulation of biofilm matrices rich in extracellular DNA. Future experiments that quantify the proportion of LPS modified by positive charges, which would increase polymyxin E resistance, in the presence and absence of DNase in wild-type HUMC1 and its BfmR-deficient derivative would lend insight into the operative mechanism. Biofilms also may provide a direct physical barrier to polymyxin E, independent of their effect on gene expression. This has been supported by *in vitro* findings in other bacterial genera (Mah and O’Toole, 2001; Kirby et al., 2012).

The pleiotropic effects of BfmR may contribute to an overall phenotype of increased intrinsic antimicrobial resistance, which may be mediated by several mechanisms, including regulation of outer membrane protein expression, LPS modification, biofilm production, peptidoglycan modification (Geisinger et al., 2018), and increased carbapenemase/oxacillinase production. A more complete delineation of these mechanisms holds the promise of developing new treatment approaches for XDR *A. baumannii*. Our transcriptosome analysis in the meropenem sensitive strain AB307-0294 demonstrated that BfmR increased expression nearly 10-fold of *bla* OXA-51, which encodes for a chromosomal oxacillinase (Supplementary Table 1); increased expression of *bla* OXA-51 has been shown to increase carbapenem resistance (Evans and Amyes, 2014). However, a mutant construct we generated in AB307-0294 in which *bla* OXA-51 was disrupted had a similar sensitivity to meropenem as its wild-type parent (MBC 2 μg/mL for both strains). These unpublished data demonstrate that BfmR mediated increased expression of *bla* OXA-51 did not affect resistance to meropenem in this strain. It remains unclear whether these findings are applicable to carbapenem resistant strains, to strains that possess multiple carbapenemases/oxacillinases, or if BfmR regulates additional carbapemenases/oxacillinases that may be present. Further, an additional area for future exploration involves the relative roles of BfmR and activating insertion sequences (e.g., IS *aba*1) on *bla* OXA-51 expression (Geisinger et al., 2018).

Our study has some limitations. First, only a single strain was studied. Next, the statistical analysis of the RNAseq defined transcriptome was underpowered due to a single sample for each strain. As a result, we focused our functional analysis on genes that were >10-fold up- or down-regulated. Additional RNAseq studies with a larger sample size could more fully delineate the BfmR regulon. However, the value of such studies for the genes of interest identified in this report would be limited, since RNA expression as measured by RNAseq was confirmed by qPCR for *oprB* and *mapA*. Lastly, ratios could not be calculated for 105 genes for which RNA was not detected in AB307-0294, in AB307.70, or both strains (Supplementary Table 1, marked in magenta). Likely explanations include less than optimal sequencing depth, the small sample size, or lack of expression. Nonetheless, it is likely that some of these genes are significantly regulated by BfmR (e.g., *csuB*). Since carbapenems are hydrophilic and transported via porins in other Gram-negative organisms (García-Sureda et al., 2011; Wozniak et al., 2012; Richardot et al., 2015), it seems logical that outer membrane proteins would enable entry into the bacterial cell in *A. baumannii*. Although our study of the BfmR-regulated porins OprB and aquaporin (Omp33-36) has not revealed a role in carbapenem resistance to date, several additional outer membrane proteins regulated by BfmR have yet to be evaluated (Supplementary Table 1). Another BfmR-regulated porin, termed CarO, has previously been implicated in antimicrobial resistance in HUMC1 (Siroy et al., 2005; Vila et al., 2007; Lu et al., 2009; Catel-Ferreira et al., 2011; Lee et al., 2011; Mussi et al., 2011; Cardoso et al., 2016; Ambrosi et al., 2017). We have shown that BfmR significantly upregulates the expression of *carO* transcription 28-fold in log phase in MM in the clinical isolate *A. baumannii* AB307-0294 (ABBFA_RS04540, Supplementary Table 1). We did create an isogenic CarO mutant in AB307-0294, which, in contrast to HUMC1, is sensitive to both polymyxin E and meropenem. However, disruption of *carO* did not affect the activity of meropenem and polymyxin E against AB307-0294 (data not shown). Therefore, we elected not to study the role of CarO in HUMC1. Studies on biofilm specifically addressed antimicrobial susceptibilities in the context of reducing the mass of young (24-h) biofilms. It remains to be seen whether these results would be applicable to more mature biofilms. Further, since the decrease in biofilm production mediated by DNase (73%) and BfmR deletion (92.9%), are not equivalent, one cannot differentiate with certainty whether the increased sensitivity to polymyxin E, with the loss of BfmR (MBC decreased from 6 μg/mL to 1 μg/mL) compared to DNase treatment (MBC decreased from 6 μg/mL to 3 μg/mL), was due to the 19.9% greater loss of biofilm formation in the former or could be attributed to biofilm independent factor(s) regulated by BfmR itself. However, the increased sensitivity of HUMC1 to polymyxin E observed with the loss of BfmR (MBC decreased from 2 μg/mL to 0.75 μg/mL) when grown under planktonic conditions is consistent with the possibility that BfmR regulated factors that do not affect biofilm formation are contributory to increased resistance to polymyxin E in addition to the unequivocal effect of biofilm. Likewise, one cannot exclude with certainty whether the modest increase in sensitivity of HUMC1 to meropenem observed with the loss of BfmR under biofilm inducing conditions was due to the greater loss of biofilm (19.9% difference) versus biofilm independent factors that increase resistance to meropenem. However, the increased sensitivity of HUMC1 to meropenem observed with the loss of BfmR (from 64 μg/mL to 25 μg/mL) when grown under planktonic conditions suggests that BfmR regulated factors that do not affect biofilm formation are more likely to be contributory to increased resistance to meropenem. However, regardless, the effect of the biofilm in decreasing antimicrobial activity was greater for polymyxin E than meropenem. Lastly, our study has focused on the role of porins in antimicrobial resistance in an extensively drug-resistant isolate of *A. baumannii*. It is possible that increased carbapenem resistance could be apparent with porin inactivation in more susceptible isolates, but not in an XDR strain. However, since BfmR upregulates OprB and aquaporin expression while conferring increased antibiotic resistance in both susceptible (AB307-0294) and XDR (HUMC1) isolates, any such effect, if present, would be predicted to be minor.

In summary, we have not yet been able to identify BfmR-regulated outer membrane proteins that increase antimicrobial resistance to carbapenems, though such a mechanism seems likely based on their mechanism of entry in other bacterial pathogens (Papp-Wallace et al., 2011). Studies in this report demonstrate that BfmR-mediated enhancement of biofilm formation increases resistance to polymyxins, but has little, if any effect, on meropenem activity. This observation warrants further investigation since it suggests that for strains susceptible to carbapenems this class of antimicrobials may be more efficacious than others in the management of *A. baumannii* infections with biofilm formation such as foreign body infections. It also suggests the possibility that polymyxins may not be the drug of choice in this setting if other more efficacious alternatives are available. Data on the activity of additional classes of antimicrobials against *A. baumannii* under biofilm inducing conditions would be an important area for future studies.

## Data Availability Statement

The original contributions presented in this study are publicly available. These data can be found here: https://www.ncbi.nlm.nih.gov/sra/PRJNA666998.

## Author Contributions

CM and TR conceived the study. CM, UM, GT, SC, and TR performed the experiments and analyzed the data. CM, UM, and TR wrote the manuscript. All authors edited and approved the manuscript.

## Conflict of Interest

The authors declare that the research was conducted in the absence of any commercial or financial relationships that could be construed as a potential conflict of interest.

## Footnotes

1 https://www.ncbi.nlm.nih.gov/assembly/GCA_000021145.1

2 https://blast.ncbi.nlm.nih.gov/Blast.cgi
