# Supplementary material for: An Evaluation of BfmR-Regulated Antimicrobial Resistance in the Extensively Drug Resistant (XDR) Acinetobacter baumannii Strain HUMC1
Source: Front Microbiol. 2020 Oct 29;11:595798. doi: 10.3389/fmicb.2020.595798 (PMC7658413; doi:10.3389/fmicb.2020.595798)
Supplement: Supplementary file 1 [file Image_1.PDF]

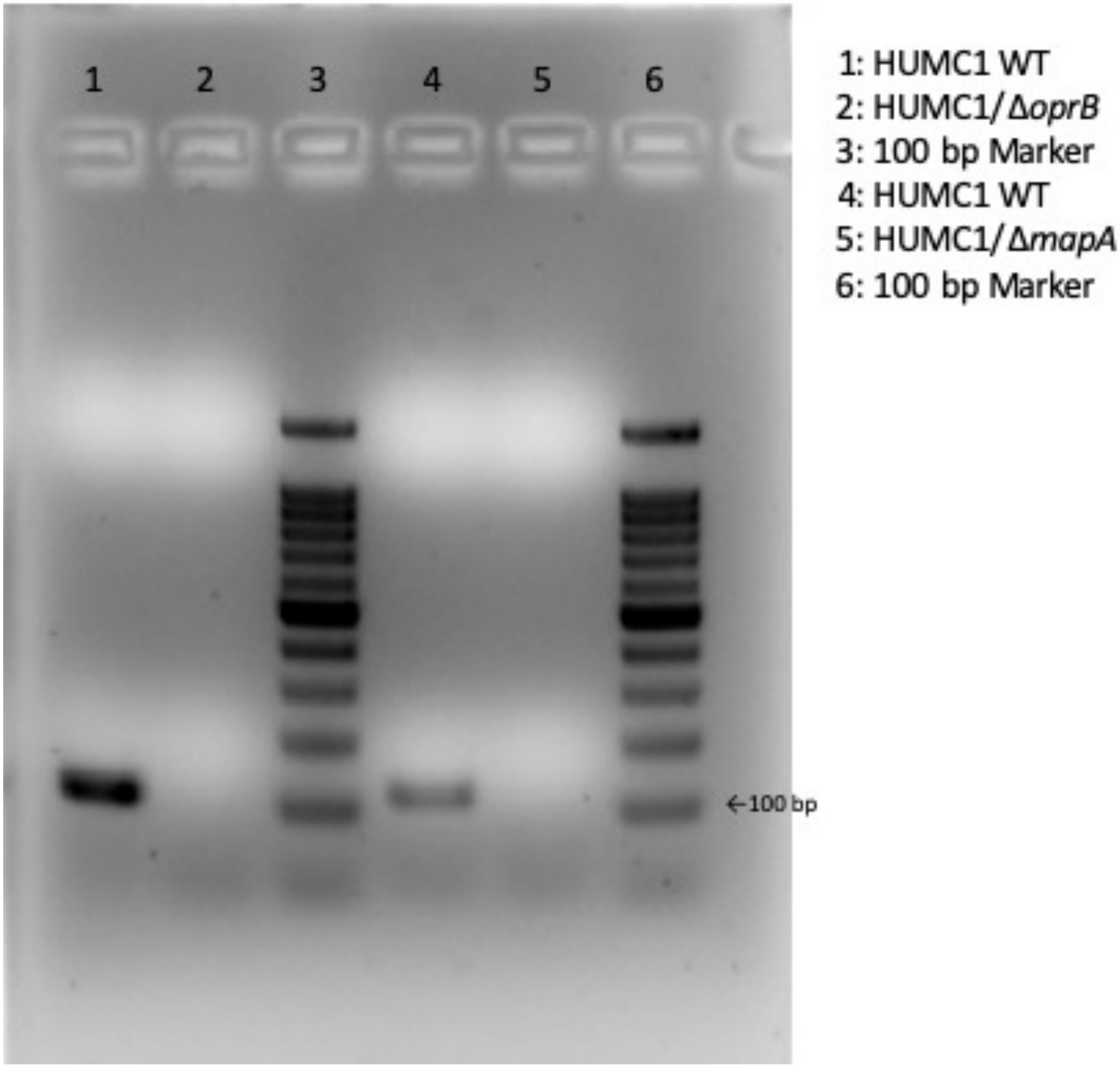

Supplemental Figure 1: RT-PCR confirmation of the absence of *oprB* and *mapA* transcript in *A. baumannii* HUMC1*ΔoprB* and HUMC1*ΔmapA* in comparison to the WT. Primers used for the PCR are same that were used for the Q-PCR (1221/22 for *oprB* WT 105 bp; 1318/19 for *mapA* WT 120 bp).
