# Supplementary material for: An Evaluation of BfmR-Regulated Antimicrobial Resistance in the Extensively Drug Resistant (XDR) Acinetobacter baumannii Strain HUMC1
Source: Front Microbiol. 2020 Oct 29;11:595798. doi: 10.3389/fmicb.2020.595798 (PMC7658413; doi:10.3389/fmicb.2020.595798)
Supplement: Supplementary file 4 [file Table_3.DOCX]

**Supplemental Table 3: Primers used in this study**

|  | Sequence | Gene |
| --- | --- | --- |
| 1189 | ATGAATAAATATTTTGCTGAATTCCTAGGTACGTTTTGGCTAGTCTTTGGTGGTTGTGGTAGCGCAGTTTTAGCCGCAGCTTTCCCTGAACTTGGTATTGGCTTTGCAGGTGTAGCCCTTGCCTTTAAAGCCACGTTGTGTCTCAAAATC | *mapA* deletion primer forward |
| 1204 | AGGTGTAGCGGTGAAATGCG | 16S rDNA forward  (Q-PCR) |
| 1205 | GGGTATCTAATCCTGTTTGCTCCC | 16S rDNA reverse  (Q-PCR) |
| 1215 | TTGACCCAAATGGTTCTTGGATGTTGGGTGATTGGAACGGGCAACGTACAGCGTTGCAAGCACAAGGTTATGATTTTTCTTTTGGATATACCGGTGAATATGCCGGTATTTTAGATTCCAAACAAAAAAGCCACGTTGTGTCTCAAAATC | *oprB* deletion primer forward |
| 1216 | TTAGAATGCAGTTGAGAACTTAATACCGCCTACCCAAGTGTTATCACCATTTTTTAATGCACCAACATGACGAACATATTGCACATTTGGACGAATAGTTAGCCAGTTAGTGGCATGAATACCATACATTATTCCCTCCAGGTCAGGCGC | *oprB* deletion primer reverse |
| 1221 | AGGTTTGCTCAATCAACGTCC | *oprB* forward (Q-PCR) |
| 1222 | CGGTGTCGTATTCTTTGGCT | *oprB* reverse (Q-PCR) |
| 1260 | TTACTTTTGAAAGCCCAAAAGTAAACAAAAGGCTTTGTTGATCAGAGGGTACATCCATGTACCCCTGCTCAACAGGTGACATCCATGTCACCTTCCGCGATAGTAGATTGAGTCTAAGAAGAGTTACATTATTCCCTCCAGGTCAGGCGC | *mapA* deletion primer reverse |
| 1318 | CTGCTGGCTTCGCTCCTATTGC | *mapA* forward (Q-PCR) |
| 1319 | CAGCAAAGAACGCAACACCTGTACT | *mapA* reverse (Q-PCR) |
